# Anti-tumor necrosis factor therapy improves insulin resistance, beta cell function and insulin signaling in active rheumatoid arthritis patients with high insulin resistance

**DOI:** 10.1186/ar3874

**Published:** 2012-06-12

**Authors:** Ilias Stagakis, George Bertsias, Stylianos Karvounaris, Melina Kavousanaki, Dimitra Virla, Amalia Raptopoulou, Dimitrios Kardassis, Dimitrios T Boumpas, Prodromos I Sidiropoulos

**Affiliations:** 1 Rheumatology, Clinical Immunology and Allergy, University of Crete, Medical School, Voutes 1, Heraklion, 71003, Greece; 2 Department of Internal Medicine, University of Crete, Medical School, Voutes 1, Heraklion, 71003, Greece; 3 Department of Basic Sciences, University of Crete Medical School, Voutes 1, Heraklion, 71003, Greece; 4 Developmental & Functional Biology, Institute of Molecular Biology and Biotechnology, Foundation for Research and Technology - Hellas, Nikolaou Plastira 100, Heraklion, 70013, Greece

## Abstract

**Introduction:**

Prevalence of insulin resistance and the metabolic syndrome has been reported to be high in rheumatoid arthritis (RA) patients. Tumor necrosis factor (TNF), a pro-inflammatory cytokine with a major pathogenetic role in RA, may promote insulin resistance by inducing Ser^312 ^phosphorylation (p-Ser^312^) of insulin receptor substrate (IRS)-1 and downregulating phosphorylated (p-)AKT. We examined whether anti-TNF therapy improves insulin resistance in RA patients and assessed changes in the insulin signaling cascade.

**Methods:**

Prospective study of RA patients receiving anti-TNF agents (infliximab, *n *= 49, adalimumab, *n *= 11, or etanercept, *n *= 1) due to high disease activity score in 28 joints (DAS28 > 5.1). A complete biochemical profile was obtained at weeks 0 and 12 of treatment. Insulin resistance, insulin sensitivity and pancreatic beta cell function were measured by the Homeostasis Model Assessment (HOMA-IR), the Quantitative Insulin Sensitivity Check Index (QUICKI) and the HOMA-B respectively. Protein extracts from peripheral blood mononuclear cells were assayed by western blot for p-Ser^312 ^IRS-1 and p-AKT. RA patients treated with abatacept (CTLA4.Ig) were used as a control group for insulin signaling studies.

**Results:**

At study entry, RA patients with high insulin resistance (HOMA-IR above median) had significantly higher mean DAS28 (*P *= 0.011), serum triglycerides (*P *= 0.015), and systolic blood pressure levels (*P *= 0.024) than patients with low insulin resistance. After 12 weeks of anti-TNF therapy, patients with high insulin resistance demonstrated significant reduction in HOMA-IR (*P *< 0.001), HOMA-B (*P *= 0.001), serum triglycerides (*P *= 0.039), and increase in QUICKI (*P *< 0.001) and serum HDL-C (*P *= 0.022). Western blot analysis in seven active RA patients with high insulin resistance showed reduction in p-Ser^312 ^IRS-1 (*P *= 0.043) and increase in p-AKT (*P *= 0.001) over the study period. In contrast, the effect of CTLA4.Ig on p-Ser^312 ^IRS-1 and p-AKT levels was variable.

**Conclusions:**

Anti-TNF therapy improved insulin sensitivity and reversed defects in the insulin signaling cascade in RA patients with active disease and high insulin resistance. The impact of these biochemical changes in modifying cardiovascular disease burden in active RA patients remains to be seen.

## Introduction

Insulin resistance is a key feature of obesity, metabolic syndrome, and type 2 diabetes mellitus (T2DM). Insulin signaling is a complex process; binding of insulin to its receptor induces both auto-phosphorylation and phosphorylation of tyrosine residues on insulin receptor substrate (IRS) proteins, the most prominent being IRS-1 and IRS-2, thus initiating the intracellular signaling cascade [1,2]. IRS-1 and IRS-2 mediate their metabolic effects through the phosphatidyl-inositol 3-kinase (PI-3K) pathway, which results in activation of AKT and other downstream effector molecules. IRS-1 may be more closely linked to glucose homeostasis, whereas IRS-2 is primarily involved in lipid metabolism [3]. Insulin signaling may also activate the mitogen activated protein kinase (MAPK) isoforms ERK1 and ERK2, through Grb/Sos and ras. This pathway mediates the mitogenic and pro-inflammatory responses of insulin signaling while it does not affect glucose homeostasis [4]. In obese patients with insulin resistance, the pathways leading to PI-3K activation are blocked, whereas the MAPK pathway remains active or even hypersensitive [5].

Inflammation and insulin resistance are closely linked and inflammatory cytokines such as tumor necrosis factor (TNF), interleukin (IL)-6, IL-1 and IL-8 may inhibit insulin signaling by multiple mechanisms [6]. TNF induces phosphorylation of IRS-1 at serine instead of tyrosine residues and promotes insulin resistance [7,8]. Both IL-6 and TNF may inhibit the transcription of *IRS-1 *and glucose transporter (*GLUT)-4 *genes, thus reducing glucose transport and enhancing insulin resistance in obese patients [9].

Patients with rheumatoid arthritis (RA) are at increased risk for cardiovascular disease [10] independently of traditional vascular risk factors [11]. Cohort studies have demonstrated increased prevalence of metabolic syndrome in patients with RA, correlating with disease activity and markers of atherosclerosis [12-14]. RA patients are also at increased risk for T2DM compared with non-rheumatic controls (adjusted hazard ratio 1.5) [15], and pancreatic beta cell function is associated with disease activity and cumulative dose of glucocorticoids [14].

Observational studies suggest that anti-TNF therapy improves disease activity and may reduce cardiovascular events in RA patients (age-sex adjusted rate ratio 0.46) [16,17]. This effect is thought to be mediated by reduction in insulin resistance and metabolic syndrome components demonstrated in patients treated with TNF blockade [18-22]. However, the results of the aforementioned studies are limited by the inclusion of a small number of RA patients and the lack of any mechanistic insights to the molecular effects of TNF blockade on insulin signaling.

To this end, we set out a 12-week prospective study in patients with RA, who were receiving anti-TNF agents due to active disease, to assess changes in insulin resistance/sensitivity, serum lipoproteins, and activation of the insulin receptor signaling cascade. Anti-TNF therapy resulted in significant improvement in insulin resistance/sensitivity and pancreatic beta cell function in RA patients with high baseline insulin resistance. Importantly, our studies in peripheral blood mononuclear cells from RA patients demonstrated reduction in IRS-1 serine phosphorylation and increase in AKT phosphorylation following anti-TNF therapy, both modifications linked to improved glucose homeostasis.

## Materials and methods

### Patients and treatment

We prospectively studied patients with RA who were started on anti-TNF agents due to high disease activity, defined by a disease activity score in 28 joints (DAS28) > 5.1 despite treatment with disease-modifying anti-rheumatic drugs (DMARDs) [23]. Patients fulfilled the American College of Rheumatology 1987 classification criteria for RA [24]. Patients aged < 18 years or with history of DM were excluded. Anti-TNF agents (infliximab, adalimumab or etanercept) were given at approved standard doses. The dosage of background DMARDs and glucocorticoids, as well as of anti-hypertensive and lipid-lowering treatments remained stable during the study period. Detailed medical and drug history was obtained, and the use of tobacco was recorded. Disease activity (DAS28) was assessed at baseline and after 12 weeks of treatment [25]. Coronary heart disease was defined as presence of any of the following: angina pectoris, myocardial infarction or coronary revascularization procedures (bypass grafting, percutaneous coronary intervention). As a control group, we consecutively enrolled seven patients with RA who were started on abatacept (CTLA4.Ig), another class of biologic therapy which targets T-cell costimulation rather than TNF, due to high disease activity (DAS28 > 5.1). All patients signed informed consent forms, and the study protocol was approved by the University Hospital of Heraklion Ethics Committee (13/12/99).

### Assessment of insulin resistance/sensitivity and other metabolic parameters

Blood was drawn after 12-hour overnight fasting. Serum concentrations of total cholesterol (TC), high-density lipoprotein cholesterol (HDL-C), and triglycerides (TG) were measured using an automated chemistry analyzer (Olympus AU-600, Tokyo, Japan). Low-density lipoprotein cholesterol (LDL-C) was calculated according to the Friedewald formula except for samples with serum TG > 400 mg/dl (4.5 mmol/L). Apolipoprotein (apo)-A, apo-B and lipoprotein a (Lpa) were analyzed by immunonephelometry (BN II analyzer, Eschborn, Germany). Plasma glucose concentration was determined by standard enzymatic colorimetric assay (Roche Diagnostics, Mannheim, Germany). C-reactive protein (CRP) was measured by immune nephelometry (BN II analyzer, Eschborn, Germany) and erythrocyte sedimentation rate (ESR) by an automated analyzer (Ves-matic 20, Florence, Italy). Serum insulin was determined by chemiluminescent microparticle immunoassay (ARCHITECT Insulin Reagent Kit, 8K41, Abbott Diagnostics, Lake Forest, Illinois, USA)

While the hyperinsulinemic euglycemic clamp technique is the gold standard for evaluating insulin sensitivity, the Homeostasis Model Assessment (HOMA) for insulin resistance and the Quantitative Insulin Sensitivity Check Index (QUICKI) are widely used as noninvasive surrogate markers of insulin resistance and sensitivity, respectively [26,27]. Pancreatic beta cell function was assessed by the HOMA for beta cell function (HOMA-B) [28]. We used the National Cholesterol Education Program (NCEP) Adult Treatment Panel (ATP) III criteria for metabolic syndrome [29].

### Isolation of peripheral blood mononuclear cells (PBMCs) and protein extracts

PBMCs were isolated by Ficoll-Histopaque (Sigma-Aldrich, St. Louis, MO, USA) density-gradient centrifugation of heparinized venous blood. PBMCs were lysed with RIPA buffer containing 50 mM Tris-HCl, pH 7.2, 150 mM sodium chloride, 1% NP-40, 12 mM sodium deoxycholate, 3 mM sodium dodecyl sulfate, 4 mM sodium azide, 0.57 mM phenylmethysulfonyl fluoride and complete protease inhibitor cocktail. Protein concentrations were determined by Bradford protein assay (Bio-Rad laboratories, Inc, Hercules, CA, USA).

### Western blot analysis

Proteins were separated by 8% sodium dodecyl sulphate-polyacrylamide gel electrophoresis (SDS-PAGE) and transferred to nitrocellulose membrane (Whatman, Schwerte, Germany). Membranes were probed overnight with anti-phospho-IRS1-Ser^312 ^(Upstate, Lake Placid, NY, USA) and anti-phospho-Akt-Ser^473 ^(Cell Signalling, Frankfurt, Germany) monoclonal antibodies. Blots were also probed with anti-*β*-actin (Santa Cruz, CA, USA) to control for total protein content. The secondary antibodies horseradish peroxidase (HRP)-conjugated anti-mouse IgG and anti-rabbit IgG were used (Santa Cruz, CA, USA). Enhanced chemiluminescence (ECL) from Thermo Scientific (Vantaa, Finland) was used for detection, and densitometry analysis was performed with Image J program.

### Statistical analysis

Results are expressed as the mean and 95% confidence interval (95% CI) or as proportions. Parameters showing abnormal distribution were logarithmically transformed. In a *post-hoc *analysis, we used the median HOMA-IR value (3.18) to define high insulin resistance, and we compared the recorded variables in RA patients with high versus low insulin resistance using the independent samples *t*-test (continuous variables) or the chi-squared test (categorical variables). The Pearson's test was used for bivariate correlation analysis. Logistic regression (backward selection) was further performed to calculate odds ratios (ORs) for high versus low HOMA-IR according to demographic and lifestyle (gender, age, tobacco use), metabolic (obesity, hypertension, use of statins, metabolic syndrome), and clinical parameters, including the DAS28, health assessment questionnaire (HAQ), CRP, and RA treatment modality (glucocorticoids, methotrexate). Changes in recorded parameters between baseline and the end of the 12-week study period were analyzed by paired *t*-test or the Wilcoxon signed ranks test as appropriate. To address whether the effect of anti-TNF treatment on insulin resistance is confounded by changes in disease activity, we performed repeated measures analysis of variance (ANOVA) entering HOMA-IR values at baseline and at 12 weeks after treatment as dependent variables (within-subject variables), the insulin resistance group (high versus low baseline HOMA-IR) as grouping factor (between-subject variable), and the change in DAS28 score as a covariate. *P *values (two-tailed) < 0.05 were considered statistically significant. SPSS software version 18.0 (SPSS, Inc) was used for the statistical calculations.

## Results

### Patients' clinical characteristics at baseline

We prospectively studied 61 patients with RA (43 women) of mean age 60 years and mean disease duration 7.2 years (Table 1). All patients had active disease with mean DAS28 score 5.8 and they were started on one of the following anti-TNF agents: infliximab (*n *= 49), adalimumab (*n *= 11), or etanercept (*n *= 1). Fifty six patients (92%) were on single (*n *= 36) or combination (*n *= 20) therapy with DMARDs, including methotrexate (*n *= 42), leflunomide (*n *= 15), and hydroxychloroquine (*n *= 17). Thirteen patients were on glucocorticoids. Patients treated with abatacept had comparable demographic and clinical characteristics and metabolic profiles to patients treated with anti-TNFagents (data not shown).

**Table 1:** Baseline characteristics of the rheumatoid arthritis patients included in the study*

		Insulin resistance		
	All patients (*n *= 61)	Low † (*n *= 31)	High † (*n *= 30)	*P *value #
Age (years)	60 (57, 63)	58 (54, 63)	62 (58, 67)	0.157
Gender (% female)	70	74	63	0.360
Disease duration (years)	7.2 (5.6, 9.3)	6.9 (4.8, 10.1)	7.5 (5.2, 10.9)	0.727
				
DAS28	5.8 (5.5, 6.1)	5.4 (5.1, 5.8)	6.3 (5.8, 6.7)	0.011
HAQ	1.0 (0.8, 1.2)	0.9 (0.6, 1.1)	1.1 (0.9, 1.4)	0.129
C-reactive protein (mg/dl)	1.4 (0.8, 2.1)	1.2 (0.2, 2.1)	1.7 (0.7, 2.6)	0.134
ESR (mm/hr)	35 (29, 40)	33 (24, 42)	36 (28, 44)	0.559
				
Insulin (μIU/ml)	13.5 (10.4, 17.4)	7.0 (5.1, 9.5)	26.7 (21.0, 33.8)	< 0.001
FBG (mg/dl)	96 (89, 103)	87 (80, 93)	107 (95, 119)	< 0.001
HOMA-IR **§**	3.2 (2.4, 4.3)	1.5 (1.1, 2.0)	7.0 (5.2, 9.4)	< 0.001
HOMA-B **§**	159 (122, 207)	123 (77, 195)	210 (168, 263)	0.013
QUICKI	0.33 (0.31, 0.34)	0.36 (0.34, 0.39)	0.29 (0.28, 0.30)	< 0.001
				
TC (mg/dl)	221 (206, 235)	226 (204, 247)	216 (195, 236)	0.471
HDL-C (mg/dl)	54 (50, 57)	56 (52, 61)	51 (46, 57)	0.095
LDL-C (mg/dl)	137 (125, 150)	144 (126, 162)	131 (114, 148)	0.278
TC/HDL-C	4.3 (3.9, 4.6)	4.1 (3.7, 4.5)	4.4 (3.9, 4.9)	0.399
LDL-C/HDL-C	2.7 (2.4, 2.9)	2.6 (2.3, 3.0)	2.7 (2.3, 3.1)	0.868
Triglycerides (mg/dl)	151 (132, 171)	129 (105, 152)	175 (144, 206)	0.015
Lpa (mg/dl)	12.8 (9.5, 17.1)	10.1 (6.6, 15.4)	16.4 (10.9, 24.8)	0.130
apoA (mg/dl)	162 (155, 169)	168 (158, 177)	156 (146, 166)	0.079
apoB (mg/dl)	99 (92, 107)	100 (88, 112)	99 (89, 109)	0.762
apoB/apoA	0.63 (0.58, 0.69)	0.61 (0.52, 0.70)	0.66 (0.57, 0.74)	0.364
Homocysteine (μmol/l)	17.5 (15.7, 19.3)	17.8 (15.1, 20.5)	17.2 (14.6, 19.9)	0.584
				
Body weight (kg)	75 (71, 79)	73 (68, 78)	77 (7, 83)	0.203
Body mass index (kg/m^2^)	29.1 (27.5, 30.7)	28.6 (26.2, 30.9)	29.7 (27.4, 32.1)	0.371
Waist circumference (cm)	101 (97, 105)	100 (94, 106)	102 (97, 107)	0.207
SBP (mmHg)	127 (123, 131)	125 (118, 132)	130 (125, 134)	0.024
DBP (mmHg)	77 (75, 79)	78 (74, 81)	76 (73, 79)	0.672
				
Metabolic syndrome (%) **	41	19	63	0.001
Statin use (%)	19	13	24	0.333
Tobacco use (%)	19	23	14	0.506
Coronary heart disease (%)	14	10	17	0.472
Hypertension (%)	61	58	63	0.795
Antihypertensive therapy (%)	46	48	43	0.799
MTX (%)	69	65	73	0.582
MTX dose (mg/week)	14.8 (14.0, 15.7)	15.9 (14.7, 17.1)	13.9 (12.7, 15.0)	0.033
GC (%)	21	19	23	0.762
GC dose (mg/day)	8.8 (5.6, 12.1)	7.9 (5.3, 10.5)	9.6 (3.0, 16.2)	0.705

* Except where indicated otherwise, values are the mean (95% confidence interval)

† The median Homeostasis Model Assessment for insulin resistance (HOMA-IR) value at baseline was used to assign patients into low (HOMA-IR ≤ 3.18, *n *= 31) or high (HOMA-IR > 3.18, *n *= 30) insulin resistance group

# *P *value for the comparison between low and high insulin resistance groups

**§ **Statistical comparisons were performed using log transformed values. Values represent the geometric mean

** According to the Adult Treatment Panel (ATP) III criteria for the definition of the metabolic syndrome

DAS28: disease activity score of 28 joints; HAQ: health assessment questionnaire; ESR: erythrocyte sedimentation rate; FBG: fasting blood glucose; QUICKI: Quantitative Insulin Sensitivity Check Index; TC: total cholesterol; HDL-C: high-density lipoprotein cholesterol; LDL-C: low-density lipoprotein cholesterol; Lpa: lipoprotein a; apo: apolipoprotein; SBP: systolic blood pressure; DBP: diastolic blood pressure; MTX: methotrexate; GC: glucocorticoids.

### Association of clinical and metabolic parameters with insulin resistance in RA patients

We used the median HOMA-IR at baseline to assign patients to the low (HOMA-IR ≤ 3.18, *n *= 31) and high (HOMA-IR > 3.18, *n *= 30) insulin resistance group. Patients in the high insulin resistance group had higher mean disease activity (*P *= 0.011), fasting serum insulin (*P *< 0.001), plasma glucose (*P *< 0.001), triglycerides (*P *= 0.015), and systolic blood pressure levels (*P *= 0.024) than patients with low insulin resistance (Table 1). Metabolic syndrome was more frequent in RA patients with high insulin resistance (63% versus 19% in those with low insulin resistance, *P *= 0.001). In accordance with previous reports [30], we found significantly increased pancreatic beta cell function, assessed by the HOMA-B index, in RA patients with high insulin resistance.

By using a cut-off of HOMA-IR > 2.0 [31], 74% (*n *= 45) of RA patients were classified as having insulin resistance. Patients with HOMA-IR > 2.0 at baseline had higher serum insulin (*P *< 0.001), HOMA-B (*P *< 0.001), and triglycerides (*P *= 0.014), and lower QUICKI (*P *< 0.001) than those with HOMA-IR ≤ 2.0. RA patients with insulin resistance had a trend for higher DAS28 (*P *= 0.105) and significantly higher HAQ (*P *= 0.037). Fasting plasma glucose levels were not significantly different between the two groups, although 61% in patients with insulin resistance versus 6% in those without insulin resistance had metabolic syndrome (*P *= 0.001) (data not shown).

HOMA-IR correlated with DAS28 (*r *= 0.31, *P *= 0.016) and fasting triglycerides (*r *= 0.38, *P *= 0.003), and HOMA-B correlated with body mass index (BMI) (*r *= 0.28, *P *= 0.035) (data not shown). In multivariate regression analysis, presence of the metabolic syndrome and DAS28 score > 6.86 (75^th ^percentile) were independent predictors for high insulin resistance with ORs of 10.8 (95% CI 2.8 to 42.6, *P *= 0.001) and 6.4 (95% CI 1.2 to 32.9, *P *= 0.027), respectively. BMI > 30 kg/m^2 ^was associated with an OR of 3.8 (95% CI 1.1 to 12.4, *P *= 0.030) for increased pancreatic beta cell function.

### Anti-TNF therapy improves insulin resistance/sensitivity, pancreatic beta cell function, and metabolic parameters in patients with active RA and high insulin resistance

Patients with high baseline insulin resistance demonstrated significant improvement in DAS28 (*P *= 0.013), HAQ (*P *= 0.012), and CRP (*P *= 0.007) after 12 weeks of treatment with anti-TNF agents (Table 2). A similar trend was observed in patients with lower baseline insulin resistance, yet only reduction in HAQ was statistically significant (*P *= 0.018). Anti-TNF therapy resulted in significant improvement in HOMA-IR (*P *< 0.001), QUICKI (*P *< 0.001), and HOMA-B (*P *= 0.001) only in RA patients with high baseline insulin resistance (Table 2). In these patients, significant decreases in fasting plasma glucose (*P *= 0.016), serum insulin (*P *< 0.001), and serum triglycerides (*P *= 0.039), and increase in serum HDL-C (*P *= 0.022) levels were observed.

**Table 2:** Effect of anti-TNFα treatment on clinical and metabolic profile in rheumatoid arthritis RA patients with low and high baseline insulin resistance*

	Insulin resistance			
	
	Low (*n *= 31)	***P *value**†	High (*n *= 30)	*P *value
DAS28	-0.52 (-1.20, 0.16)	0.127	-0.57 (-1.01, -0.13)	0.013
HAQ	-0.23 (-0.42, -0.05)	0.018	-0.26 (-0.45, -0.06)	0.012
ESR	-3.04 (-15.3, 9.20)	0.611	-1.04 (-7.91, 5.84)	0.760
C-reactive protein #	-0.69 (-1.91, 0.53)	0.147	-0.83 (-1.72, 0.05)	0.007
TC	-5.92 (-20.0, 8.21)	0.395	8.63 (-6.53, 23.8)	0.253
Triglycerides	12.3 (-16.7, 41.3)	0.390	-24.5 (-47.7, -1.31)	0.039
HDL-C	0.75 (-3.25, 4.75)	0.701	4.21 (0.65, 7.78)	0.022
LDL-C	-9.78 (-20.2, 0.64)	0.065	9.15 (-3.94, 22.2)	0.163
TC/HDL-C	-0.08 (-0.35, 0.18)	0.511	-0.12 (-0.44, 0.19)	0.421
LDL-C/HDL-C	-0.14 (-0.31, 0.03)	0.110	0.01 (-0.25, 0.27)	0.929
Lpa #	-4.38 (-9.31, 0.55)	0.147	0.25 (-7.72, 8.22)	0.200
apoA	-6.12 (-17.3, 5.03)	0.262	5.64 (-3.94, 15.2)	0.236
apoB	-2.99 (-13.0, 6.99)	0.534	2.09 (-4.81, 8.99)	0.537
Homocysteine #	-2.66 (-5.88, 0.57)	0.122	2.53 (-0.35, 5.41)	0.039
FBG	1.08 (-1.02, 1.18)	0.117	-19.7 (-34.8, -4.61)	0.016
HOMA-IR #	1.87 (-0.34, 4.09)	0.197	-5.68 (-8.86, -2.50)	< 0.001
HOMA-B #	-130 (-375, 116)	0.446	-148 (-244, -46)	0.001
QUICKI #	-0.01 (-0.03, 0.01)	0.264	0.07 (0.04, 0.09)	< 0.001
Insulin #	5.80 (-1.12, 12.7)	0.301	-21.7 (-33.8, -9.62)	< 0.001

Values are the mean (95% confidence interval)

* The median Homeostasis Model Assessment for insulin resistance (HOMA-IR) value at baseline was used to assign patients into low (HOMA-IR ≤ 3.18, *n *= 31) or high (HOMA-IR > 3.18, *n *= 30) insulin resistance group

† Paired *t*-test for comparison of values between baseline and after 12 weeks of anti-TNF treatment

# Statistical comparisons were performed using log transformed values

DAS28: disease activity score of 28 joints; HAQ: health assessment questionnaire; ESR: erythrocyte sedimentation rate; TC: total cholesterol; HDL-C: high-density lipoprotein cholesterol; LDL-C: low-density lipoprotein cholesterol; Lpa: lipoprotein a; apo: apolipoprotein; FBG: fasting blood glucose; QUICKI: Quantitative Insulin Sensitivity Check Index.

We repeated the analysis in RA patients with baseline HOMA-IR ≤ 2.0 (*n *= 16) or > 2.0 (*n *= 45). Anti-TNF treatment reduced DAS28 (*P *= 0.007), HAQ (*P *= 0.002), and CRP (*P *= 0.002) in patients with insulin resistance but not in those with HOMA-IR ≤ 2.0 (data not shown). TNF blockade resulted in significant reduction in serum insulin (*P *< 0.001) and improvements in HOMA-IR (*P *= 0.001), HOMA-B (*P *= 0.001), and QUICKI (*P *= 0.001) in patients with HOMA-IR > 2.0. In contrast, there were no significant changes in serum triglycerides (*P *= 0.058), HDL-C (*P *= 0.101), homocysteine (*P *= 0.312) and blood glucose (*P *= 0.166). These findings suggest that the metabolic effects of anti-TNF treatment may be more pronounced in RA patients with very high insulin resistance levels compared to those with low-level or no insulin resistance.

We next examined whether changes in insulin resistance/sensitivity and pancreatic beta cell function correlated with changes in RA disease activity. Significant improvements in the HOMA-IR, QUICKI, and HOMA-B indices were found in RA patients with high baseline insulin resistance irrespective of their clinical response according to the European League Against Rheumatism (EULAR) criteria (data not shown). In addition, repeated measures ANOVA revealed a significant effect of anti-TNF treatment (*F *test = 11.89, *P *= 0.001) and of the interaction of anti-TNF treatment with baseline insulin resistance group (*F *test = 24.68, *P *< 0.001) on HOMA-IR. Conversely, the interaction of anti-TNF treatment with the change in DAS28 had no significant impact on HOMA-IR (*F *test = 0.06, *P *= 0.810), suggesting that the effect of anti-TNF treatment on insulin resistance is not confounded by improvement in disease activity.

### Anti-TNF therapy reduces IRS-1 phosphorylation at Ser^312^

**Figure 1:**
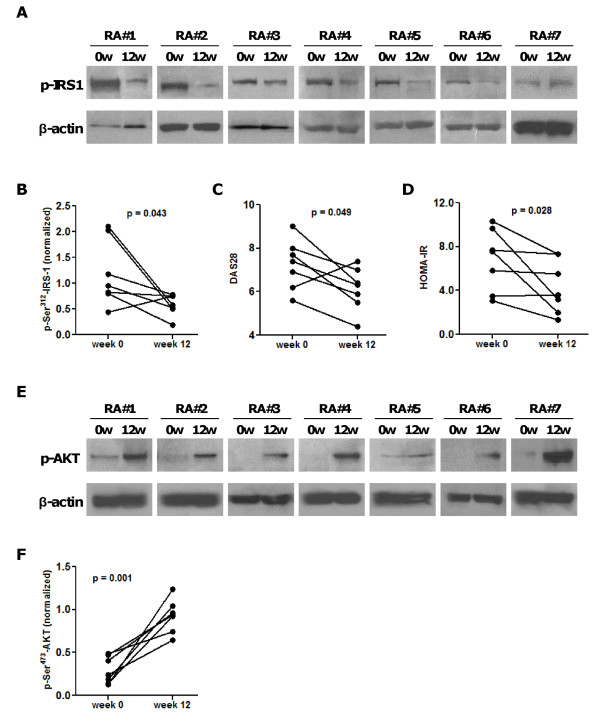
**Anti-TNF treatment decreases insulin receptor substrate (IRS)-1 Ser^312 ^phosphorylation and increases AKT phosphorylation in patients with active rheumatoid arthritis (RA) and high insulin resistance**. **(A) **Seven patients with active RA received anti-TNF treatment for 12 weeks. Protein extracts from PBMCs were analyzed by western blotting for the Ser^312^-phosphorylated form of IRS-1 at weeks 0 and 12 of treatment. All but one patient demonstrated decrease in IRS-1 phosphorylation at Ser^312^. **(B) **Quantitative analysis (protein densitometry) showed that median phosphorylated IRS-1 normalized for *β*-actin levels were reduced from 0.96 to 0.57 (arbitrary units) (Wilcoxon signed ranks test, *P *= 0.043). **(C) **Decrease in disease activity following anti-TNF treatment (from median DAS28 7.4 to 6.3, *P *= 0.049). **(D) **Improvement in insulin resistance following anti-TNF treatment (from median HOMA-IR 7.6 to 3.6, *P *= 0.028). **(E) **Protein extracts from peripheral blood mononuclear cells (*n *= 7 RA patients) were analyzed by western blotting for Ser^473^- phosphorylated AKT at weeks 0 and 12 of anti-TNF therapy. **(F) **Phosphorylated AKT levels, normalized for β-actin, increased in all RA patients from a median 0.24 (arbitrary units) at week 0 to 0.94 at week 12 (*P *= 0.001).

TNF may compromise insulin signaling by induction of serine 312 (Ser^312^) phosphorylation of IRS-1 [7,32]. Based on our findings of improved insulin resistance in patients with active RA following treatment with anti-TNF agents, we examined whether this effect is associated with altered IRS-1 phosphorylation. We used PBMCs, which provide a useful tool to determine alterations in the insulin signaling cascade *in vivo *as they include all of the signaling proteins present in insulin target tissues [33,34]. We purified total protein extracts from PBMCs of seven randomly selected RA patients at weeks 0 and 12 of anti-TNF therapy, and we quantified IRS-1 Ser^312 ^phosphorylation by western blot. At study entry, all patients had active disease with median DAS28 7.4 (range 5.6 to 9.0), and high insulin resistance with median HOMA-IR 7.6 (range 3.1 to 10.3). At week 12, all but one patient demonstrated reduction in IRS-1 phosphorylation (Figure 1A). Phosphorylated IRS-1 normalized for β-actin protein expression was significantly reduced from 0.96 (arbitrary units) at week 0 to 0.57 at week 12 (Wilcoxon signed ranks test, *P *= 0.043) (Figure 1B). Both DAS28 and HOMA-IR improved over the study period (Figures 1C, D). We found no correlation between longitudinal changes in HOMA-IR and IRS-1 Ser^312 ^phosphorylation levels.

### Anti-TNF therapy increases AKT phosphorylation

IRS-1 mediates its metabolic effects through the PI-3K pathway which results in phosphorylation of AKT and other downstream molecules [32]. TNF-induced insulin resistance involves inhibition of AKT through a ceramide-activated protein-phosphatase (PP) 2A [35]. Individuals with insulin resistance display reduced insulin stimulation of the PI-3K pathway [5], and insulin-stimulated AKT kinase activity is reduced in patients with T2DM [36,37]. Therefore, we measured phosphorylated AKT levels in protein extracts from PBMCs of RA patients at weeks 0 and 12 of anti-TNF therapy. Phosphorylated AKT levels, normalized for β-actin, increased in all seven RA patients (Figure 1E) from a median 0.24 at week 0 to 0.94 at week 12 (*P *= 0.001) (Figure 1F). We found no correlation between longitudinal changes in HOMA-IR or IRS-1 Ser^312 ^phosphorylation and AKT phosphorylation levels.

### Improvement in RA disease activity following therapy with CTLA4.Ig does not correlate with changes in insulin resistance, IRS-1 and AKT phosphorylation

To assess the specificity of the effect of TNF blockade on insulin resistance, we prospectively studied seven RA patients (median age 63 years, median disease duration 13 years) with high disease activity (median DAS28 5.92) treated with abatacept (CTLA4.Ig), a biologic agent that targets T-cell co-stimulation. All patients were receiving DMARDs (five on methotrexate, two on leflunomide), and three patients were receiving low-dose glucocorticoids. At baseline, three patients had low insulin resistance (median HOMA-IR 1.16, range 0.87 to 1.91), and four patients had high insulin resistance (median HOMA-IR 5.34, range 3.45 to 8.85).

**Figure 2:**
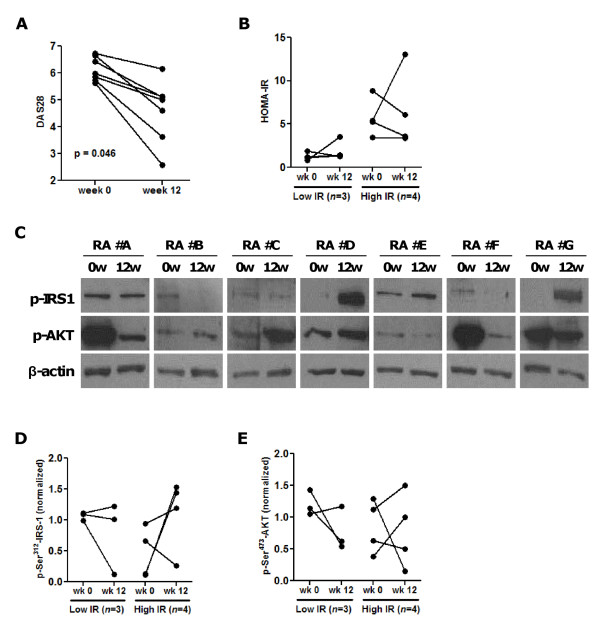
**Variable effect of CTLA4.Ig (abatacept) treatment on insulin receptor substrate (IRS)-1 and AKT phosphorylation in patients with rheumatoid arthritis (RA)**. **(A) **Improvement in disease activity in RA patients (*n *= 7) following treatment with abatacept for 12 weeks, from median disease activity score in 28 joints (DAS28) 5.9 to 4.8 (*P *= 0.046, Wilcoxon signed ranks test). **(B) **Treatment with abatacept had variable effects on the Homeostasis Model Assessment for insulin resistance (HOMA-IR) in RA patients with low or high baseline insulin resistance. **(C) **Protein extracts from periperhal blood mononuclear cells of the RA patients were analyzed by western blotting for the Ser^312 ^and Ser^473^- phosphorylated forms of insulin resistance substrate (IRS)-1 and AKT, respectively, at baseline and after 12 weeks treatment with abatacept. **(D) **Quantitative analysis of p-Ser^321 ^IRS-1 normalized to β-actin expression showed no significant change following treatment with abatacept. **(E) **Variable trend in p-AKT levels from baseline to week 12 of treatment with abatacept in RA patients with low or high baseline insulin resistance.

At week 12 of abatacept treatment, disease activity was reduced in all seven RA patients (Wilcoxon signed ranks test, *P *= 0.046) (Figure 2A). Insulin resistance followed a variable trend, and HOMA-IR increased in three patients (two with low baseline insulin resistance), remained stable in one patient, and decreased in three patients (two with high baseline insulin resistance) (Figure 2B). Similarly, HOMA-B was not significantly altered during the study period (data not shown).

To examine the effect of CTLA4.Ig treatment on the insulin signaling cascade, we measured the levels of Ser^312^-phosphorylated IRS-1 and phosphorylated AKT (p-AKT) in protein extracts from PBMCs (Figure 2C). We found no significant change in p-Ser^321 ^IRS-1 or in p-AKT levels in RA patients with either low or high baseline insulin resistance (Figure 2D, E). We found no correlation between changes in HOMA-IR and IRS-1 or AKT phosphorylation levels.

## Discussion

We evaluated the outcome on insulin resistance and other metabolic parameters among 61 patients with active RA disease, after 12 weeks of treatment with anti-TNF agents. In a subset of patients, we also examined the effect of TNF blockade on the phosphorylation status of IRS-1 and AKT, which are major mediators of insulin signaling. Our findings suggest that treatment with anti-TNF agents may improve insulin resistance and sensitivity in RA patients with active disease and high insulin resistance, and this effect correlates with reduced levels of the insulin-resistant Ser^312^-phosphorylated form of IRS-1 and increased levels of activated/phosphorylated AKT.

Insulin resistance constitutes a major mechanism in the pathophysiology of metabolic syndrome [38]. In our cohort of patients with active RA, those with high insulin resistance had significantly higher fasting plasma glucose, serum triglycerides, and systolic blood pressure levels than their counterparts in the low insulin resistance group. Accordingly, patients with metabolic syndrome had an OR of 10.8 for high insulin resistance, independent of the effect of other parameters.

In patients with RA, insulin resistance has been shown to correlate both with anthropometric parameters (abdominal obesity) and high disease activity [13,14]. We found that RA patients with high insulin resistance had significantly higher DAS28 values than those with low insulin resistance, and in multivariate analysis, patients in the upper quartile of disease activity (DAS28 > 6.86) had more than six times increased risk (OR 6.4) for high insulin resistance. This is in agreement with our previous result of increased prevalence of metabolic syndrome in RA patients with moderate or high disease activity (OR 9.2 for DAS28 > 3.2) [12], and corroborates the link between inflammation and insulin resistance in chronic inflammatory disorders.

Previous studies have demonstrated that treatment with TNF antagonists improves insulin sensitivity in RA [18-22]. We further corroborate these results by examining the metabolic effects of anti-TNF therapy in a larger cohort of 61 patients with active RA. Among patients with high insulin resistance at baseline, treatment with anti-TNF agents for 12 weeks resulted in significant improvement in insulin sensitivity as evidenced by reduction in the HOMA-IR and increase in the QUICKI indices. Of note, serum triglycerides were reduced by an average 24.5 mg/dl, and HDL-C levels were increased by an average 4.2 mg/dl. Our findings agree with those of Kiortsis, *et al*. [20] who reported significant decrease in HOMA-IR and increase in QUICKI among patients with RA and ankylosing spondylitis in the highest tertile of insulin resistance, suggesting that this subset of patients may benefit most from anti-TNF therapy. In accordance with the results of previous studies [18-20,22], we found no correlation between changes in HOMA-IR and fasting insulin levels with improvements in disease activity or CRP level.

We found that treatment with anti-TNF agents resulted in significant reduction in the HOMA-B index of pancreatic beta cell function in RA patients with high baseline insulin resistance. This could be explained by the therapy-driven reduction in the inflammatory burden of the disease and the insulin resistance levels, thus resulting in reduced compensatory beta cell hyperfunction. Whether this effect could lead to preservation of beta cell function in the long term is currently unknown. Intriguingly, a recent epidemiological study found that among patients with RA or psoriasis, the adjusted risk of new-onset DM was lower for individuals on TNF inhibitor (adjusted hazard ratio 0.62) compared with initiation of other non-biologic DMARDs [39].

TNF may impair insulin signaling at the level of the IRS proteins, and the Ser^307 ^(or Ser^312 ^in humans) residue in IRS-1 has been identified as a site for the inhibitory effects of TNF, with p38 MAPK and inhibitor kB kinase being involved in the phosphorylation of this residue [7,8,32]. TNF mediates insulin resistance by inhibition of AKT activity through a ceramide-activated protein-phosphatase (PP) 2A [35]. Since anti-TNF therapy significantly reduced HOMA-IR in patients with active RA and high insulin resistance, we examined the extent to which the aforementioned biochemical effects are affected by TNF inhibition. Our western blot assays demonstrated significant reduction in p-Ser^312 ^IRS-1 and increase in p-AKT levels, associated with reduction in HOMA-IR in patients with active RA and high baseline insulin resistance. These alterations were consistent in all but one patient in our study and their magnitude did not correlate with the degree of improvement in disease activity or insulin resistance. To our knowledge, this is the first demonstration that anti-TNF therapy may improve insulin resistance in patients with active RA by reversing defects in the phosphorylation/activation status of the insulin signaling pathway.

To determine whether the observed improvements in insulin resistance and phosphorylation status of IRS-1 and AKT are directly linked to TNF blockade or are secondary to reduction in disease activity and inflammation, we studied seven patients with RA, who received abatacept (CTLA4.Ig) due to high disease activity. Patients treated with abatacept did not demonstrate reduction in HOMA-IR levels over the 12-week study period despite improvement in disease activity, nor did they display any consistent alterations in p-Ser^312 ^IRS-1 and p-AKT levels. It should be noted, however, that these observations are limited to a small number of patients and further documentation in a larger cohort will be required. Moreover, it may be argued that the duration of the study was too short to capture the metabolic effects of abatacept treatment, or that we did not evaluate changes in other molecules implicated in insulin resistance such as expression of the protein-tyrosine phosphatase (PTP)-1B or the glucose transporter (GLUT)-4 [32].

Although our results suggest a beneficial effect of anti-TNF treatment on insulin signaling in RA patients with high insulin resistance, the clinical importance of such treatment in reducing risk of cardiovascular disease in the long term remains to be determined. In the interim, clinical decisions for initiation of anti-TNF or other biologic agents in RA should be based on disease activity and severity indices [40]. To this end, according to the existing EULAR recommendations, 'adequate control of disease activity is necessary to lower the cardiovascular disease risk' in RA patients [41]. Consequently, anti-TNF agents may be preferred as first-line biologic treatment in patients with active RA and high inflammatory burden, and metabolic abnormalities or insulin resistance.

## Conclusions

In summary, we found that 12 weeks of treatment with anti-TNF agents may improve insulin resistance in patients with active RA and high insulin resistance. Treatment with anti-TNF was shown to restore the phosphorylation status of Ser^312^-IRS-1 and AKT, which are important mediators in the insulin signaling cascade. The impact of these biochemical changes in modifying the burden of cardiovascular disease in patients with chronic inflammatory arthritis remains to be seen.

## Abbreviations

ANOVA: analysis of variance; apo-A: apolipoprotein; ATP: Adult Treatment Panel; BMI: body mass index; CI: confidence interval; CRP: C-reactive protein; DAS28: disease activity score of 28 joints; DBP: diastolic blood pressure; DMARD: disease-modifying anti-rheumatic drug; ESR: erythrocyte sedimentation rate; EULAR: European League Against Rheumatism; FBG: fasting blood glucose; GC: glucocorticoids; HAQ: health assessment questionnaire; HDL-C: high-density lipoprotein cholesterol; HOMA-IR: Homeostasis Model Assessment for insulin resistance; HRP: horseradish peroxidase; IL: interleukin; IRS: insulin receptor substrate; LDL-C: low-density lipoprotein cholesterol; Lpa: lipoprotein a; MAPK: mitogen activated protein kinase; NCEP: National Cholesterol Education Program; OR: odds ratio; PBMC: peripheral blood mononuclear cell; PI-3K: phosphatidyl-inositol 3-kinase; PP: protein-phosphatase; QUICKI: Quantitative Insulin Sensitivity Check Index; RA: rheumatoid arthritis; SBP: systolic blood pressure; SDS-PAGE: sodium dodecyl sulphate-polyacrylamide gel electrophoresis; T2DM: type 2 diabetes mellitus; TC: total cholesterol; TG: triglycerides; TNF: tumor necrosis factor.

## Competing interests

The authors declare that they have no competing interests.

## Authors' contributions

IS performed PBMC separation and western blotting. GB contributed to study design, statistical analysis, and drafting the manuscript. SK performed patients' data acquisition and took care of the patients. MK and DV helped with blood sampling and performed western blotting. AR took care of the patients and also helped with patients' data acquisition. DK contributed to the design of the experimental part of the study. DTB and PIS supervised the study, drafted and revised the manuscript. All authors read and approved the final manuscript.
